# Clinical Significance of *TNFRSF1A*36T/C Polymorphism in Cachectic Patients with Chronic Heart Failure

**DOI:** 10.3390/jcm10051095

**Published:** 2021-03-05

**Authors:** Grzegorz Sobieszek, Tomasz Powrózek, Aneta Skwarek-Dziekanowska, Teresa Małecka-Massalska

**Affiliations:** 1 Department of Cardiology, 1st Military Clinical Hospital with the Outpatient Clinic, 20-080 Lublin, Poland; anetask@gmail.com; 2 Department of Human Physiology, Medical University of Lublin, 20-059 Lublin, Poland; tmalecka@gmail.com

**Keywords:** chronic heart failure, cachexia, *TNFRSF1A*, cardiac cachexia

## Abstract

**Introduction:** One of the main factors contributing to the development of nutritional deficits in chronic heart failure (CHF) patients is the systemic inflammatory process. Progressing inflammatory response leads to exacerbation of the disease and could develop into cardiac cachexia (CC), characterized by involuntary weight loss followed by muscle wasting. The aim of this study was to assess the relationship between rs767455 (36 T/C) of the *TNFRSF1A* and the occurrence of nutritional disorders in CHF patients with cachexia. **Materials and Methods:** We enrolled 142 CHF individuals who underwent cardiac and nutritional screening in order to assess cardiac performance and nutritional status. The relationship between *TNFRSF1A* rs767455 genotypes and patients’ features was investigated. **Results:** A greater distribution of the TT genotype among cachectic patients in contrast to non-cachectic individuals was found (TT frequencies of 62.9% and 37.1%, respectively; *p* = 0.013). We noted a significantly lower albumin concentration (*p* = 0.039) and higher C-reactive protein (CRP) levels (*p* = 0.019) in patients with the TT genotype. Regarding cardiac parameters, CHF individuals bearing the TT genotype demonstrated a significant reduction in ejection fraction (EF) (*p* = 0.033) in contrast to other genotype carriers; moreover, they had a significantly higher concentration of N-terminal prohormone of brain natriuretic peptide (NT-proBNP) in the blood (*p* = 0.018). We also noted a lower frequency of TT genotype carriers among individuals qualified as grades I or II of the New York Heart Association (NYHA) (*p* = 0.006). The multivariable analysis selected the TT genotype as an unfavorable factor related to a higher chance of cachexia in CHF patients (Odds ratio (OR) = 2.56; *p* = 0.036). **Conclusions:** The rs767455TT genotype of *TNFRSF1A* can be considered as an unfavorable factor related to a higher risk of cachexia in CHF patients.

## 1. Introduction

Chronic heart failure (CHF) is a clinical syndrome characterized by systemic inflammation and immune system activation [1]. The increase in the number of newly diagnosed CHF cases is related to a rising life expectancy (patient’s age >70 years) and diastolic heart failure [2,3]. Inflammation plays a crucial role in chronic heart failure. Traditional serum markers are of limited value in CHF. One of them that has an important impact is the tumor necrosis factor (TNF)-alpha binding to two specific receptors: TNFR1 and TNFR2. TNFR1 exerts antiviral and cytotoxic activity and stimulates fibroblast proliferation. That is why it can have an impact on the myocardial remodeling process. TNFR1s have been found to be strongly connected with CHF complications. One of these complications includes cardiac cachexia (CC). The main factor contributing to the development of cachexia is the ongoing inflammatory process. The mechanism of cachexia progression in patients with CHF is based on reduced myocardial perfusion where inflammation plays a crucial role [4,5]. One of the genes associated with the development of the inflammatory response is *TNFRSF1A.* The protein encoded by this gene belongs to the TNF receptor family. This major receptor for TNF-alpha activates NF-kappaB, mediates apoptosis, and regulates inflammation [6].

The effect of single nucleotide polymorphisms (SNPs) rs2229094, rs1041981, and rs1799964 of the *TNF/lymphotoxin* gene cluster and *TNFRSF1A* was investigated in relation to the outcome of Hepatitis C Virus (HCV) infection. Genetic polymorphism in the *TNF/lymphotoxin* and *TNFRSF1A* genes was demonstrated to have an impact on the susceptibility and chronicity of HCV infection among the Chinese Han population [7]. In another study, genetic polymorphisms in TNF-alpha receptors illustrated a variable response to treatment with TNF-alpha inhibitors in patients with Crohn’s disease [8]. *TNFRSF1A:rs767455* (36T/C) was found to have an impact on anti-TNF-alpha clinical outcomes among patients with Crohn’s disease. It was associated with a higher incidence of infections with *Mycobacterium avium* subsp. *paratuberculosis* (MAP).

To our knowledge, there are no studies on the correlation between the *TNFRSF1A* gene status and the development of nutritional disorders in patients with CHF. The aim of this study was to assess the relationship between SNPs (36T/C) of the *TNFRSF1A* gene and the occurrence of nutritional disorders in CHF patients with cachexia.

## 2. Material and Methods

### 2.1. Study Group

There were 142 CHF individuals (mean age: 72 ± 13 years) enrolled in the study group. All individuals were patients of the Clinic of Cardiology and Internal Medicine, Department of Cardiology, Military Hospital in Lublin, Poland, in the period between 2013 and 2015. The European Society of Cardiology (ECS) criteria were applied for the CHF diagnostic purposes and supplemented by patients’ clinical screenings and echocardiographic assessments (ejection fraction (EF); left ventricular end-diastolic and end-systolic diameters (LVEDd and LVESd); left anterior descending artery (LAD); tricuspid annular plane systolic excursion (TAPSE); right ventricular outflow tract (RVOT), followed by laboratory tests (serum concentration of N-terminal prohormone of brain natriuretic peptide (NT-proBNP), lipidogram, creatinine and hemoglobin concentration). To assess the extent of the disease, the New York Heart Association (NYHA) functional classification was applied. The following inclusion criteria for the study participants were defined: (1) age >18 years and Polish ethnicity; (2) signed informed consent to participate in the study; (3) a lack of metallic implants or an implanted cardioverter defibrillator. The exclusion criteria were the following: (1) acute coronary syndrome; (2) extreme renal failure; (3) recent coronary artery bypass grafting (within the last 6 months); (4) hyper- or hypothyroidism. Characteristics of the study group are presented in Table 1. The Bioethical Commission in the Medical University of Lublin approved the study protocol (No. of consent: KE-0254/64/2017).

### 2.2. Nutritional Assessment and Cachexia Detection

We adapted Evans’s criteria for cachexia detection, as follows: a weight loss of ≥5% or more in 12months or less in the presence of underlying illness, plus three of the following criteria: decreased muscle strength, fatigue, anorexia, low fat-free mass index, abnormal biochemistry (increased inflammatory markers: C-reactive protein >5.0 mg/L), anemia (hemoglobin <12 g/dL), and a low concentration of serum albumin (<3.2 g/dL)) [9]. The observation duration was 15 months, and throughout this period we measured the parameters. Based on the above mentioned inflammatory and nutritional deficiencies, our patients fulfilled the criteria of cachexia.

The nutritional screening included anthropometric measurements (body weight, BMI), followed by a subjective global assessment (SGA) questionnaire. The parameters reflecting nutritional status and the body composition were derived from bioelectrical impedance analysis (BIA) and included fat mass (FM) and fat-free mass (FFM). Additionally, the phase angle (PA) (at a 50 kHz frequency) and capacitance of cell membrane (Cm) values were derived from the BIA. Similar conditions of the BIA measurements were provided for all study participants. The ImpediMed bioimpedance analysis SFB7 BioImp v1.55 device (Pinkenba, QLD, Australia) was used to measure the parameters reflecting the body composition.

We collected 5 mL of peripheral blood from each patient participating in the study. The samples were stored at −80 °C until further laboratory analysis. DNA isolation was performed by the column method using a dedicated kit according to the manufacturer’s recommendations (DNA Blood Mini Kit, Qiagen, Toronto, ON, Canada). The genotyping reaction based on the Real-Time PCR technique was performed on a StepOnePlus device (Applied Biosystems, Foster City, CA, USA) according to the manufacturer’s protocol (using a Genotyping Master Mix and TaqMan probes specific for *TNFRSF1A* SNP: rs1799964) (Thermo Fisher Scientific, Waltham, MA, USA).

### 2.3. Statistical Analysis

MedCalc 15.8 computer software was used for statistical analysis purposes (MedCalc Software, Belgium). Data distribution of the studied parameters was checked by the Shapiro–Wilk test. Based on the results, either parametric or non-parametric statistical tests were applied. Differences between the values of the studied parameters independent of the studied SNP were recorded with the use of ANOVA or the ANOVA Kruskal–Wallis test. To analyze the differences in the values of the studied parameters between the cachectic and non-cachectic groups, either the Student *t*-test (parametric) or the Mann–Whitney *U* (non-parametric) test was used. To assess the cachexia probability, depending on selected demographic, clinical, and genetic factors, logistic regression (univariate analysis) and multivariable logistic regression analysis were used. Results *p* < 0.05 were considered statistically significant. The Bonferroni–Holm (BH) method was used to counteract the problem of multivariable comparisons [10]. The adjusted *p* values were introduced to the analysis and included in the tables demonstrating the study results.

## 3. Results

Based on the genotyping data, the following distribution of the *TNFRSF1A* rs767455 was obtained in the whole study group: TT in 38 patients (26.7%), CT in 64 patients (45.1%), and CC in 40 individuals (28.2%). The distribution of genotypes was consistent with the Hardy–Weinberg’s equilibrium (HWE) in the whole study group (*p* = 0.241). The distribution of studied SNPs did not depend on the clinical demographic features of CHF patients (*p* > 0.05).

First, we compared clinical demographic, cardiac, and nutritional factors in accordance with the presence of *TNFRSF1A* genotypes in CHF patients (Table 2). We noted a significantly lower albumin concentration (mean albumin concentration = 3.16 ± 0.62 g/dL (TT) vs. 3.42 ± 0.65 g/dL (CC) and 3.40 ± 0.58 g/dL (CT); *p* = 0.039) and higher C-reactive protein (CRP) levels (median CRP concentration = 12.43 mg/L (TT) vs. 4.60 mg/L (CC) and 7.70 mg/L (CT); *p* = 0.019) in patients with the TT genotype. Regarding the cardiac parameters, CHF individuals bearing the TT genotype demonstrated a significant reduction in EF (mean EF = 36 ± 14% (TT) vs. 43 ± 15% (CT) and 43 ± 15% (CC); *p* = 0.033) in contrast to other genotype carriers; moreover, they had a significantly higher concentration of NT-proBNP in the blood (median NT-proBNP concentration = 3953 pg/mL (TT) vs. 2697 pg/mL (CT) and 2077 pg/mL (CC); *p* = 0.018). We also noted a lower frequency of TT genotype carriers among individuals qualified to grades I and II of the NYHA (13.9% of TT carriers, 32.2% of CT, and 53.8% of CT individuals; *p* = 0.006). Additionally, both male and female TT carriers had significantly lower values of PA measuring at 50kHz in contrast to other genotype carriers (*p* = 0.035 and *p* = 0.032 for male and female, respectively).

In the subsequent stage of the study, we divided the CHF patients into two cohorts in relation to cachexia presence. Afterward, we compared the clinical, nutritional, and cardiac parameters between the groups (Table 3). Despite the significant differences in albumin and CRP concentration, BMI, and body weight between the patients, the cachectic ones demonstrated reduced EF (mean = 38 ± 12% and 43 ± 15%; *p* = 0.022), increased NT-proBNP (median = 4445 pg/mL and 1750 pg/mL; *p* < 0.001) and creatinine (mean = 1.36 ± 0.56 mg/dL and 1.17 ± 0.43 mg/dL; *p* = 0.033) plasma concentration. Moreover, both cachectic males and females demonstrated significantly lower values of PA at 50 kHz (*p* = 0.029 and *p* = 0.004 for men and women, respectively) in comparison with non-cachectic patients. We also recorded a greater distribution of the TT genotype among the cachectic patients in contrast to the non-cachectic individuals (TT frequencies of 62.9% and 37.1%, respectively; *p* = 0.013).

Eventually, we enrolled all studied parameters to uni- and multivariable regression analysis to identify the factors affecting the chance of cachexia in CHF individuals. In Table 4, we summarize only the factors that significantly affected cachexia in the univariate and multivariable regression models. The univariate analysis revealed a low concentration of albumin (mean <3.20 g/dL; OR = 13.6; *p* < 0.001) as the most significant factor affecting cachexia in CHF patients. Similar results were derived from multivariable regression analysis, and the patients with low albumin levels had over a sevenfold greater chance of developing cachexia (OR = 7.69; *p* = 0.005). As for the impact of *TNFRSF1A* genotypes on cachexia incidence, both the univariate (OR = 4.23; *p* = 0.019) and multivariable (OR = 2.56; *p* = 0.036) analyses selected the TT genotype as an unfavorable factor related to a higher chance of cachexia in CHF patients.

## 4. Discussion

CHF is a growing public health problem. Because the incidence of CHF increases with age, largely due to risk factors such as hypertension or the chronic inflammatory state associated with chronic diseases, the epidemic of CHF is likely to grow in the future. Chronic inflammation developing on the basis of increased cytokine production, characteristic of patients with CHF, may result in the occurrence of nutritional status disorders and, consequently, may lead to irreversible changes, such as cachexia [11]. Among many inflammatory cytokines, TNF-alpha may play an important role in the mechanisms responsible for the breakdown of muscle fibers in the ubiquitin–proteasome pathway (UPP) by activating the muscle atrophy F box (MAFbx) ligase and muscle ring-finger 1 (MuRF1) genes. Inflammation has also been confirmed to be important in the breakdown of adipose tissue. Increased production of TNF-alpha is associated with an increase in the number of fat cells undergoing apoptosis and a decrease in the rate of lipogenesis [12,13]. It is crucial to note that the role of the receptors of TNF-alpha has not been clearly assessed in the overall cardiac pathophysiology [14]. There are two forms for TNF-alpha as an inflammatory ligand: (a) the membrane-bound form and (b) the secreted form, adding another layer of complexity in the overall development of pathophysiology [15,16]. Despite the complexities of TNF-alpha signaling, the data from studies have shown that cardiomyocyte-specific expression of TNF-alpha results in depressed cardiac function that is gene dosage-dependent [17,18].

It should be noted that the available literature lacks studies directly related to the relationship between *TNFRSF1A* gene status and nutritional disorders. The available studies focus mainly on the role of this gene SNP in the course of gastrological conditions—mainly HCV and Crohn’s disease [7]. Qasem et al. found the association of SNP (TNFRSF1A:RS767455 andTNFRSF1B:RS3397) with lower expression of their corresponding genes. This has a crucial meaning concerning the initiation of treatment therapy. Patients simply should be screened for those SNPs before treatment therapy in order to have a better clinical outcome [8].

In another study, [19] the impact of polymorphism A36G of the TNF-alpha receptor 1 (TNFRSF1A + 36A/G) on plasma concentrations of PAI-1 in 163 obese 31 with metabolic syndrome (MS) and 150 lean, healthy women was tested. The researchers found that in obese women, the presence of MS significantly potentiated the elevation of sTNFR1 and PAI-1 levels observed in the TNFRSF1A + 36G/G carriers. They concluded that the association between the TNFRSF1A + 36G/G genotype and MS renders obese women more prone to activation of the TNF pathway, considering the high levels of circulating sTNFR1 and PAI-1. Another method that has been widely used in acute decompensate CHF patients as a predictor of mortality is the nutritional risk index (NRI) [20]. In our nutrition assessment, despite the SGA, BIA was included with all its restrictions, which are stable hydrate status and homogenous ethnicity.

It is difficult to compare our results to other studies, as there are no available reports regarding the correlation betweenrs767455 (36T/C) of the *TNFRSF1A* gene and the occurrence of nutritional disorders in CHF patients with cachexia. The most interesting aspects of our findings that should be highlighted are the following: (a) significantly lower albumin concentration (mean albumin concentration = 3.16 ± 0.62 g/dL (TT) vs. 3.42 ± 0.65 g/dL (CC) and 3.40 ± 0.58 g/dL (CT); *p* = 0.039) and higher CRP levels (median CRP concentration = 12.43 mg/L (TT) vs. 4.60 mg/L (CC) and 7.70 mg/L (CT); *p* = 0.019) in patients with the TT genotype; (b) patients with the TT genotype demonstrated a significant reduction in EF (mean EF = 36 ± 14% (TT) vs. 43 ± 15% (CT) and 43 ± 15% (CC); *p* = 0.033), significantly higher concentration of NT-proBNP in the blood (median NT-proBNP concentration = 3953 pg/mL (TT) vs. 2697 pg/mL (CT) and 2077 pg/mL (CC); *p* = 0.018) in contrast to other genotype carriers. What is worth mentioning is that both male and female TT carriers had significantly lower values of PA measuring at 50kHz compared to other genotype carriers (*p* = 0.035 and *p* = 0.032 for male and female, respectively). Low PA values were found in many chronic conditions, including cancers [21,22,23,24], liver cirrhosis [25], AIDS [26] and anorexia nervosa [27].

We also found that the low level of albumin among patients with CHF resulted in a sevenfold greater chance of developing cachexia (OR = 7.69; *p* = 0.005). What is more, the assessment of the *TNFRSF1A* genotype impact on the incidence of cachexia revealed, in both univariate (OR = 4.23; *p* = 0.019) and multiple (OR = 2.56; *p* = 0.036) analysis, that the TT genotype was an unfavorable factor related to a higher chance of cachexia in CHF patients. This might be a particularly useful tool for clinical specialists to use with their patients and to monitor those with the TT genotype.

Searching for new markers of inflammation involved in the development of malnutrition and cardiac cachexia, and explaining the mechanisms of their action, may allow for a more efficient diagnosis of nutritional disorders, faster implementation of nutritional treatment, and the development of new therapies. The presence of the studied SNPs in the regulatory region of the *TNFRSF1A* gene may have a significant impact on the expression of this gene and, consequently, on the production of a specific protein. The limitations of our study include the lack of assessment of the influence of diet or emerging problems with food intake. Moreover, we did not assess the influence of individual genotypes of the studied gene on its expression (and the expression of the protein it encodes). Despite these limitations, to our knowledge, this is the first study attempting at an assessment of the *TNFRSF1A:rs767455* genotype, demonstrating that it may be a useful marker in determining the risk of nutritional disorders in patients with CHF. CHF patients with low albumin levels had over a sevenfold greater chance of developing cardiac cachexia. The *TNFRSF1A: rs767455* TT genotype was found to be an unfavorable factor related to a higher risk of cachexia in CHF patients. At the molecular level, this genotype will preferably exert TNF-alpha as a proinflammatory cytokine in many tissues, which will stimulate and increase an ongoing inflammatory process.

## Figures and Tables

**Table 1:** Characteristics of the study group.

Variable		Study Group (*n* = 142)
Sex	Men	79 (55.6%)
	Women	63 (44.4%)
NYHA	I	27 (18.9%)
	II	38 (26.8%)
	III	35 (24.7%)
	IV	42 (29.6%)
SGA	A	72 (50.7%)
	B	57 (40.1%)
	C	13 (9.2%)
Diabetes mellitus	Yes	52 (36.6%)
	No	90 (63.4%)
Renal failure	Yes	49 (34.5%)
	No	93 (65.5%)
Smoking status	Smoker	87 (61.3%)
	Non-smoker	55 (38.7%)
Continuous Variables		Mean ± SD or Median (25th–75th percentile)
Age (years)		72 ± 13
Weight (kg)		83.0 ± 18.0
BMI (kg/m^2^)		29.6 ± 6.11
Albumin (g/dL)		3.39 ± 0.61
Triglycerides (mg/dL)		111 ± 64
Total cholesterol (mg/dL)		157 ± 45
HDL (mg/dL)		50 ± 18
LDL (mg/dL)		86 ± 36
Creatinine (mg/dL)		1.25 ± 0.49
Hemoglobin (g/dL)		13.1 ± 2.2
CRP (mg/L)		6.20 (2.1–35)
Systolic blood pressure (mmHg)		132 ± 24
Diastolic blood pressure (mmHg)		76 ± 14
EF%		41 ± 15
NT-proBNP (pg/mL)		2797 (1212–5145)
LVESd (cm)		4.37 ± 1.0
LVEDd (cm)		5.43 ± 1.1
LAD (cm)		4.47 ± 0.8
RVOT (cm)		3.49 ± 0.50
TAPSE (cm)		1.97 ± 1.4
PASP (mmHg)		40.27 ± 12.5

NYHA—New York Heart Associaction; SGA—subjective global assessment; BMI—body mass index; CRP—C-reactive protein; EF—ejection fraction; LVESd—left ventricular entricle endsystolic diameter; LVEDd—left ventricular enddiastolic diameter; LAD—the left anterior descending artery; RVOT—right ventricular outflow tract; TAPSE—tricuspidal annular plane systolic excursion; PASP—pulmonary artery systolic pressure.

**Table 2:** Comparison of selected variables depending on the occurrence of the *TNFRSF1A* genotypes (ANOVA *p* < 0.05; Bonferroni–Holm (BH) *p* < 0.015).

Variable		*TNFRSF1A* Genotypes (*n* = 142)			
		CC (*n* = 40; 28.2%)	CT (*n* = 64; 45.1%)	TT (*n* = 38; 26.7%)	*p*
Age (years)		71 ± 12	75 ± 13	73 ± 15	0.770
Weight (kg)		82 ± 18	83 ± 20	80 ± 15	0.593
BMI (kg/m^2^)		29.44 ± 6.53	30.18 ± 6.54	28.20 ± 4.72	0.638
FM (kg)		28.1 ± 11.7	27.9 ± 13.9	25.2 ± 10.2	0.931
FFM (kg)		54.2 ± 14.0	55.3 ± 14.1	53.7 ± 13.7	0.690
Albumin (g/dL)		3.42 ± 0.65	3.40 ± 0.58	3.16 ± 0.62	*0.039*
Triglycerides (mg/dL)		114 ± 69	110 ± 68	109 ± 46	0.936
Total cholesterol (mg/dL)		157 ± 53	157 ± 43	153 ± 43	0.894
HDL (mg/dL)		48 ± 18	49 ± 18	52 ± 20	0.377
LDL (mg/dL)		87 ± 40	85 ± 35	81 ± 34	0.664
Creatinine (mg/dL)		1.33 ± 0.51	1.22 ± 0.47	1.26 ± 0.57	0.589
Hemoglobin (g/dL)		13.6 ± 2.1	13.7 ± 2.3	13.2 ± 1.8	0.765
CRP (mg/L)		4.60 (2–17)	7.70 (2–24)	12.43 (5–35)	0.012 *
Systolic blood pressure (mmHg)		133 ± 23	133 ± 24	128 ± 25	0.533
Diastolic blood pressure (mmHg)		75 ± 14	76 ± 13	75 ± 16	0.860
EF%		43 ± 15	43 ± 15	36 ± 14	*0.033*
NT-proBNP (pg/mL)		2077 (1140–4573)	2697 (1176–4119)	3953 (1894–8982)	0.015 *
LVESd (cm)		4.50 ± 1.13	4.24 ± 0.88	4.47 ± 1.06	0.357
LVEDd (cm)		5.59 ± 1.16	5.28 ± 0.95	5.53 ± 0.96	0.266
LAD (cm)		4.46 ± 0.56	4.43 ± 0.62	4.57 ± 0.51	0.553
RVOT (cm)		3.44 ± 0.54	3.45 ± 0.54	3.59 ± 0.32	0.388
TAPSE (cm)		2.13 ± 2.23	1.84 ± 0.40	2.02 ± 1.34	0.572
PASP (mmHg)		38 ± 13	40 ± 13	42 ± 12	0.389
NYHA	I	13 (48.1%)	12 (44.4%)	2 (7.5%)	0.236
	II	8 (21.1%)	23 (60.5%)	7 (18.4%)	
	III	11 (31.4%)	14 (40%)	10 (28.6%)	
	IV	8 (19.1%)	15 (35.7%)	19 (45.2%)	
	I and II	21 (32.3%)	35 (53.8%)	9 (13.9%)	0.006 *
	III and IV	19 (24.6%)	29 (37.7%)	29 (37.7%)	
SGA	A	23 (31.9%)	37 (59.7%)	12 (8.4%)	<0.001 *
	B	16 (28.1%)	26 (45.6%)	15 (26.3%)	
	C	1 (7.7%)	1 (7.7%)	11 (84.6%)	
	A	23 (31.9%)	37 (59.7%)	12 (8.4%)	*0.022*
	B and C	17 (24.3%)	27 (38.6%)	26 (37.1%)	
Diabetes mellitus	Yes	16 (30.8%)	25 (48.1%)	11 (21.1%)	0.516
	No	24 (26.7%)	39 (43.3%)	27 (30%)	
Renal failure	Yes	14 (28.6%)	23 (46.9%)	12 (22.5%)	0.902
	No	26 (28%)	41 (44%)	26 (28%)	
Smoking status	Smoker	29 (33.3%)	38 (43.7%)	20 (23%)	0.181
	Non-smoker	11 (20%)	26 (47.3%)	18 (32.7%)	
Cm (nF)	Men	1.18 (0.78–1.97)	1.41 (0.77–1.92)	1.38 (0.71–1.72)	0.845
	Women	1.05 (0.79–2.20)	1.30 (0.94–1.64)	1.07 (0.73–1.73)	0.803
Pa (^o^)	Men	4.05 ± 1.06	4.31 ± 1.26	3.24 ± 1.02	*0.035*
	Women	4.54 ± 1.69	4.15 ± 1.11	3.26 ± 1.14	*0.032*
Z200/Z5	Men	0.870 (0.75–0.87)	0.861 (0.82–0.89)	0.864 (0.84–0.89)	0.516
	Women	0.859 (0.78–0.89)	0.853 (0.83–0.87)	0.853 (0.83–0.88)	0.930

ANOVA: *p* < 0.05; Bonferroni–Holmes correction *p* < 0.015; Italic type: significant in ANOVA test; marked by *: significant after Bonferroni correction.

**Table 3:** Comparison of selected variables depending on the occurrence of cachexia (*t*-student and Mann–Whitney *U* test *p* < 0.05; BH *p* < 0.02).

Variable		Study Group (*n* = 142)		
		Cachectic (*n* = 60)	Non-Cachectic (*n* = 82)	*p*
Age (years)		76 ± 10	72 ± 13	0.342
Weight (kg)		78 ± 20	87 ± 15	0.005 *
BMI (kg/m^2^)		28.21 ± 6.36	30.76 ± 5.66	0.017 *
FM (kg)		25.94 ± 14.52	28.05 ± 11.88	0.426
FFM (kg)		52.66 ± 14.01	55.26 ± 14.83	0.368
Albumin (g/dL)		2.99 ± 0.59	3.72 ± 0.40	<0.001 *
Triglycerides (mg/dL)		104 ± 55	117 ± 70	0.253
Total cholesterol (mg/dL)		153 ± 51	160 ± 40	0.380
HDL (mg/dL)		48 ± 19	52 ± 17	0.251
LDL (mg/dL)		85 ± 40	86 ± 33	0.771
Creatinine (mg/dL)		1.36 ± 0.56	1.17 ± 0.43	*0.033*
Hemoglobin (g/dL)		12.6 ± 2.3	13.5 ± 2.0	0.019 *
CRP (mg/L)		15.0 (6.0–34.5)	3.6 (1.7–8.3)	<0.001 *
Systolic blood pressure (mmHg)		132 ± 24	132 ± 23	0.877
Diastolic blood pressure (mmHg)		77 ± 15	74 ± 12	0.324
EF%		38 ± 12	43 ± 15	*0.022*
NT-proBNP (pg/mL)		4445 (2483–8374)	1750 (985–3323)	<0.001 *
LVESd (cm)		4.31 ± 1.06	4.43 ± 0.95	0.481
LVEDd (cm)		5.34 ± 1.12	5.50 ± 0.93	0.355
LAD (cm)		4.52 ± 0.62	4.43 ± 0.54	0.373
RVOT (cm)		3.50 ± 0.48	3.47 ± 0.52	0.790
TAPSE (cm)		1.78 ± 0.47	2.04 ± 1.61	0.212
PASP (mmHg)		43.4 ± 0.2	42.1 ± 0.1	0.645
Cm (nF)	Men	1.04 (0.82–1.70)	1.36 (0.80–1.97)	0.812
	Women	1.05 (0.84–1.40)	1.42 (1.05–1.86)	0.109
PA (°)	Men	3.47 ± 1.14	4.13 ± 1.23	*0.029*
	Women	3.46 ± 1.20	4.56 ± 0.97	0.004 *
Z200/Z5	Men	0.86 (0.83–0.89)	0.87 (0.84–0.90)	0.240
	Women	0.87 (0.85–0.90)	0.85 (0.83–0.86)	0.148
SGA	A	10 (13.9%)	62 (86.1%)	<0.001 *
	B	42 (73.7%)	15 (26.3%)	
	C	8 (61.5%)	5 (38.5%)	
*TNFRSF1A* genotype	CC	14 (30.4%)	32 (69.6%)	0.013 *
	CT	25 (41%)	36 (59%)	
	TT	22 (62.9%)	13 (37.1%)	

Student *t*-test and Mann–Whitney *U* test: *p* < 0.05; Bonferroni–Holmes correction: *p* < 0.02; Italic type: significant in ANOVA test; marked by *: significant after Bonferroni correction.

**Table 4:** Factors significantly affecting cachexia in CHF patients in the univariate and multivariable regression analyses.

Predictors (Independent Variables)	Univariate Regression Analysis		
	*p*	OR	[95%CI]
Albumin < 3.20 g/dL	<0.001	13.6	[4.35–45.40]
BMI < 24.9	0.004	4.32	[1.59–11.76]
Renal failure	0.003	3.14	[1.47–6.17]
PA < 3.15°	0.022	2.75	[1.16–6.51]
SGA B or C	<0.001	12.5	[5.23–29.98]
TT genotype of *TNFRSF1A*	0.019	4.23	[1.37–10.98]
Predictors (independed variables)	Multivariable regression analysis		
	***p***	**OR**	**[95%CI]**
Albumin < 3.20 g/dL	0.005	7.69	[1.81–30.3]
Renal failure	0.017	4.40	[1.30–14.92]
TT genotype of *TNFRSF1A*	0.036	2.56	[0.64–10.42]

Overall model fit: *p* < 0.001.

## Data Availability

Data available on request due to restrictions.
